# Prevalence of Strongyle Infection and Associated Risk Factors in Horses and Donkeys in and around Mekelle City, Northern Part of Ethiopia

**DOI:** 10.1155/2021/9430824

**Published:** 2021-07-21

**Authors:** Wossene Negash, Yosef Erdachew, Teshager Dubie

**Affiliations:** College of Veterinary Medicine, Samara University, P.O. Box 132, Samara, Ethiopia

## Abstract

**Background:**

In Ethiopia, equines serve in traction power, carting, recreation, festival packing, riding, transportation, and other activities since time immemorial. Strongyles are common equine health problems in Ethiopia though research based data on equine strongyles are limited particularly in the study areas, in and around Mekelle city. Therefore, the present study was intended to estimate the prevalence of common equine strongyles in and around Mekelle city from November 2018 to April 2019 and to assess risk factors associated with infection of strongyle parasites as well. Cross sectional design was used in this study, and the study population consisted of both donkeys and horses of all age and both sex groups. From randomly selected horses and donkeys, approximately 25 grams of faecal samples was drawn with gloved hands from rectum of study equines, labeled, and transported to laboratory for coprological examination. Flotation technique was employed to separate parasitic eggs from faeces, followed by microscopic examination for identification of strongyle eggs based on morphology. Pearson's chi-square (*χ*2) was carried out to determine association between risk factors and parasitic infection. Moreover, both bivariate and multivariate logistic regression analyses were computed to assess the strength of association of those risk factors at 95% CI and *P* < 0.05.

**Result:**

Out of 384 samples collected, 204 were found to be positive for strongyles with an overall prevalence of 53.13%. Prevalence of strongyle species in equines was also estimated to be 53% and 53.3% for donkeys and horses, respectively. Accordingly, of the six risk factors considered, only three factors (age, management type, and body condition scores) were found to influence the occurrence of strongyle infection and to be statistically significant as well.

**Conclusion:**

The higher prevalence of equine strongyles in the present study might be suggestive of urgent and coordinated actions to be in place.

## 1. Introduction

Ethiopia is endowed with immense natural resources occupying different agroecological zones and suitable environmental conditions making it a home for many livestock species and varying production systems. Recent estimated livestock population included 59.5 million cattle, 56.53 million heads of chicken, 30.70 million sheep, 30.20 million goats, 8.44 million donkeys, 2.16 million horses, 1.21 million camels, and 0.41 million mules [1]. Of the total population of equines, 364,940 are found in Tigray region [2]. Horses and donkeys are collectively named as equines. In Ethiopia, equines are used for packing, riding, carting, plowing/traction power, and transportation [3]. Moreover, equines are also used for recreation and festivity specially horses. Equines play significant roles in agricultural systems of many developing countries holding magnificent share in food security and social equity in high food insecure nations, particularly in highly inaccessible areas [4, 5].

Though equines are known by their hardy characteristics, their prominent roles are impeded by health problems [6] of which the most common challenges lead to health problems, poor reproductivity and working performances, shorter lifespan, and finally death due to infectious diseases and parasitism [7]. Endoparasites are organisms that live within the body of the host [8]. Many internal parasites can infect equines including roundworms; mainly strongyles, flukes, tapeworms, protozoans, and fly larvae damage the intestine, respiratory system, and other internal organs of equines [9–11].

Strongyle nematodes are important equine gastrointestinal parasites belonging to the Superfamily Strongyloidea, family Strongylidae, genus *Strongylus* and comprising three species: *Strongylus vulgaris*, *Strongylus edentatus*, and *Strongylus equinus.* These live as adults in the large intestine of equids [12]. Equine strongyles are grouped into small and large strongyles. Large strongyles are more pathogenic relative to their smaller counterparts. Strongylosis is a devastating health problem particularly in young horses kept on permanent pastures although severe cases may occur in adults subjected to overcrowding and poor management [13, 14]. *Strongylus vulgaris* and *Strongylus edentatus* are the commonest equine health concerns in Ethiopia [15].

Being the most pathogenic species, *Strongylus vulgaris* is known as double tooth strongyle, whereas *Strongylus edentatus* is termed as toothless strongyle and *Strongylus equinus* known as triple toothed strongyle. Relatively, the first is smaller than the second [16]. Though strongyle infections are of very much concern and economically important equine health problem in Ethiopia, generally, research works or scientific data pertaining to equine strongyles are scarcely available to date particularly in and around Mekelle city. Therefore, the objectives of the current study were to estimate the prevalence of horses and donkeys strongyle infection in and around Mekelle city and to assess associated risk factors with the disease.

## 2. Materials and Methods

### 2.1. Description of the Study Areas

This cross sectional study was conducted in and around Mekelle city, from November 2018 to May 2019. Being the capital of Tigray region, Mekelle city is located 783 km from Addis Ababa in the northern eastern part of Ethiopia. Currently, the city has 7 subcities named as Hawelti, Hadnet, Ayder, Semen, Quiha, Kedamay Weyane, and Adi Haqi. Of the 7 subcities, Hadnet, Quiha, Adi Haqi, Kedamay Weyane, and Hawelti were chosen for the current study based on convenience. Mekelle city is located at a latitude of 13^o^ 20′ 50″N and longitude of 39^o^ 32′ 38″E with an altitude range of 2150–2270 meter above sea level with arid to semiarid weather condition. The mean annual rainfall and temperature of the city were estimated at 200 mm to 11200 mm and 12.5°C to 27.5°C, respectively [17]. Tigray region covers an area of 54,548.32 km^2^ and has an estimated livestock population of 3,596,649 cattle, 1,646,752 goats, 1,064,501 sheep, 364,940 equines, 13,661 camels, and 2,570,833 poultry birds [1, 2].

### 2.2. Study Population

The study was conducted on donkeys and horses admitted to veterinary hospitals and city clinics in and around Mekelle city. The study included donkeys and horses of all age, clinically suspected, and apparently healthy groups as well as both sex groups.

### 2.3. Sampling Method and Sample Size Determination

Both the surrounding rural kebeles and Mekelle subcities were selected purposively. However, individual animal owners of study units (animals) were chosen using simple random sampling technique. To estimate the total number of animals to be included in the study, Thrusfield [18] formula was employed:

(1) $$\begin{array}{c} N=\frac{\left(1.96\right)².P\text{exp}\left(1-P\text{exp}\right)}{d{}^{2}}, \end{array}$$

where *N* is the sample size, 1.96 is the the value of “*Z*” at 95% confidence interval, *P*_exp_ is the expected prevalence, and *d* is the desired absolute precision.

By taking 95% confidence interval, 5% level of precision, and 50% expected prevalence into consideration in the above formula, the total sample size needed for the study was calculated to be 384 (200 donkeys and 184 horses). Study animals were categorized into three groups based on Loch and Bradley (1998). Accordingly, equines aged less than three years were considered as young, those aged between 3 and 10 ears as adult, and lastly equines aged more than 10 years as old.

### 2.4. Sample Collection and Laboratory Analysis Method

With gloved hands, approximately 25 grams of faecal samples was withdrawn from the rectum of restrained animals into screw capped universal sample bottle at the study sites. Following collection, each sample was uniquely labeled using each animal's identification parameters (species, age, sex, origin/site, collector's name, body condition score, and date of collection). Collected samples were packed and transported to Mekelle University Veterinary Parasitology Laboratory for immediate identification of strongyle parasites based on egg morphology.

### 2.5. Parasitological Techniques

For separation of parasite eggs from the faecal debris, flotation technique was applied. Technically, approximately 3 gm of faeces was measure and placed into glass beaker followed by addition of 40 ml flotation fluid, specifically magnesium sulfate solution. The mixture was continuously stirred using a glass rod. The dissolved suspension was then strained using tea strainer into another beaker. Then, the suspension was transferred to another test tube to an extent of meniscus formation at the top of the tube, and cover slip was gently placed over the meniscus and allowed to stand for 20 min. Finally, the cover slip to which the supernatant fluids were adhered was carefully lifted off the test tube, placed on microscopic slide, and examined under low power microscopic magnification (10x) (William, 2001). Strongyle species were identified based on oval morphology under the microscope.

In principle, flotation fluid was used to concentrate and separate eggs from most faecal debris. Since strongyle eggs have less specific gravity compared to flotation fluids (magnesium sulfate, sugar, sodium nitrate, and zinc sulfate), eggs floated and concentrated at the upper portion of the test tube containing sample mixture. On the other hand, faecal debris with higher gravity compared to the eggs concentrated at the lower portion of the test tube. Therefore, examination of the solution in the topmost layer clearly indicated the presence of strongyle parasite eggs. Hence, flotation method relies on the differences in the specific gravity of the eggs, faecal debris, and flotation solution. The flotation method is technically easy to process and faster as well. The disadvantage of most flotation techniques is that the walls of eggs and cysts can often collapse, thus hindering identification [19].

### 2.6. Data Management and Analysis

All relevant data were collected, coded, and entered precisely into Microsoft Excel application. Data were analyzed using STATA 14.0. Descriptive statistics was used to calculate prevalence and percentages whereas Pearson chi-square test (*χ*2) was employed to assess association between parasite infection and risk factors. Both bivariate and multivariate logistic regression analyses were computed to measure the strength of association between strongyle infection and risk factors with 95% CI and *P* value less than 0.05.

## 3. Results

Of 384 animals examined, 204 were found to be positive for gastrointestinal equine strongyles in both species with an overall prevalence of 53.13% (Table 1). Moreover, descriptive statistics has indicated the proportion of positive animals for species of equines. Accordingly, of the 204 animals positive for the parasites under study, 106 (53%) were donkeys and 98 (53.3%) were horses. The magnitude of parasitism was found to be approximately equivalent in both species. The study has also indicated that parasitic burdens were more in male as compared to female animals. Similarly, more parasitic intensity has been found in extensive type of management compared to semi-intensive management type. Both descriptive statistics and association analyses results are summarized in Table 1.

By applying 95% CI and *P* < 0.05, chi-square (*χ*2) analysis was computed and indicated that only three (3) factors, namely, age, body condition, and management type, were significantly associated with strongyle infection (*P* < 0.05) (Table 1). However, sex, equine species, and origin of animals had no significant association with strongyle infection (*P* > 0.05) (Table 1).

To assess the degree of influence each factor could have, keeping other factors constant, on the infection of strongyles, bivariate logistic regression analysis was computed. Accordingly, age, body condition, and management type were found to have statistically significant association with strongyle parasite infection using 95% CI and *P* value 5% (Table 2). Similarly, to assess the combined/simultaneous influence of all factors on strongyle infection, occurrence was computed employing multivariate logistic regression analysis with 95% CI and *P* < 0.05 (Table 2). In multivariate logistic regression analysis, adjusted odds ratio (AOR) was used. In age analysis, only adult equines had shown significant contribution (*P* < 0.000) to the overall significance of age impact. Adult equines were 63% less likely at risk as compared to old ones. In body condition analysis, animals with poor and medium body conditions were 6.99 times and 6.13 times more likely at risk as compared to good body conditioned animals, respectively. Similarly, management type was analyzed, and semi-intensively managed equines were 2.55 times more likely at risk as compared to extensively managed ones.

## 4. Discussion

Gastrointestinal (GI) parasite infection has a direct effect on the health and production potential of working equines, which contributes to the reduction in their work output and, ultimately, in the income of the owner and the community [20]. In this study, the overall estimated prevalence of strongyles in horses and donkeys was 53.13% (*n* = 204/384) in and around Mekelle city on the basis of coprological microscopic examination results. The current study finding closely agreed with previous reports of Ashenafi et al. [21], who reported 47.4% in horse and donkeys in and around Kombolcha town, Ethiopia, and similarly Pandey and Eysker [22], who reported a prevalence of 48% in donkeys in Zimbabwe. On the other hand, the current finding was found to be lower as compared to the reports of Molla et al. [23, 24], who reported a prevalence of 64.61%, 100%, 99%, and 100% for Menz Keya Gerbil District, East Shewa, and Adaa and Akaki of East Shewa, respectively. The nutritional status can affect the host immunity level, which means if the study animals access good nutrition, they would have enhanced immunity that can protect the host from parasite infection. On the other hand, if the study animals are malnutrioned, their immunity status would be decreased so that the study animals can be easily affected [25].

Moreover, the present finding was observed to be higher than the findings of Singh et al. [26], who reported a prevalence of 27.33% in Punjab and India, and Disassa et al. [25], who reported 5.37% in and around Dangila town, Ethiopia. These observed variations could be attributed to a difference in parasite epidemiology, managemental difference, lack of donkey sanctuary, absence of regular deworming service, and restriction on pasture grazing. In the current study, the prevalence of strongyles in horses and donkeys was estimated to be 53.3% and 53%, respectively, and the degree of strongyle infection seems to be slightly higher in horses compared to donkeys with no statistical difference (*P* > 0.05).

The current finding closely agreed with the reports of Ma and wubit [12], who reported the prevalence of strongyle infections to be 44.55% in donkeys and 48.2% in horses. However, the current study revealed a higher prevalence at species level than Disassa et al. [25], who reported 5.82% in donkeys and 4.92% in horses in and around Dangila town. On the other hand, the current study result was lower than the reports of Getachew et al. [24] from East Shewa-Adaa that revealed 100% and 99% in donkeys and in horses and Hassan et al. [27], who reported a higher prevalence of 99.15% in Sudan. Moreover, the current result was lower than that reported by Feseha et al. [20] (100% in donkeys) and those reported by Tolla et al. [28] in and around Gondar, Ethiopia, Tesfu et al. [29] in and around Hawassa town, and Ra and Etaferahu [11] in South Wollo zone (87.81%, 76%, 70.8% in donkeys and 66.67%, 64.9%, 58.5% in horses, respectively). In addition, the current study finding was lower as compared to previous reports by Mezgebu et al. [30], who reported 66.7% prevalence in Gondar Town, and Uslu and Guçlu [31], who reported 100% in Konya, Turkey. The observed variations could be attributable to agroecological difference, parasite load in the study areas, and equine population in the study sites. Furthermore, differences could be associated with hygienic conditions, regular deworming services, and reduced parasitism [26].

Statistically, the age of study animals and strongyle infection were significantly associated (*P* < 0.05). Similar outcome was observed by Dryden et al. [32] and Ibrahim et al. [33], who reported statistically significant difference between age groups with higher prevalence in adult and lower prevalence in young animals. The agreement of the findings could be associated with study design, technique applied, sample size, geographic conditions, work load, and management difference or husbandry practices.

The present study also estimated prevalence of equine strongylosis to be 45% in females and 54% in males with no significant difference (*P* > 0.05). Similar phenomenon was reported by Fikru et al. [34, 35]. The absence of significant association between the parasite infection and sex might be due to communal grazing system, biological difference, anthelmintic treatment services, and husbandry practices. However, in previous study findings, Bereket et al. and Basaznew et al. [36, 37] have reported significant association between parasitic infection and sex. The significant association observed in these studies might be due to the fact that females are managed for breeding purpose and kept in home whereas male equines are used for packing purposes and carry wood a long distance to market places usually crossing different agroecological areas which might increase the probable exposure of males to strongyle infection as compared to their female counterparts.

The current study has also indicated a statistically significant difference (*P* < 0.05) between body condition scores and strongyle infections with infection rates of 81.82%, 45.18%, and 43.83% for good, medium, and poor body condition scores, respectively. The current study has reported a slightly higher prevalence related to body condition score compared to Mangassa and Tafese [38], who reported a significant association between body condition and parasite infection in and around Batu town with prevalence of 59.26%, 43.17%, and 30.6% in poor, medium, and good body condition scores, respectively. However, the current study finding was lower as compared to the findings of Tesfu et al. [29], who reported 72.5%, 71.6%, and 70.7% in poor, medium, and good body condition scores, respectively. Similarly, significant difference was reported by FAO and Francisco et al. [39, 40]. The present study has also reported prevalence of equine strongyle infection in study sites of 50%, 47%, 60%, 63%, and 51% for Hadnet, Quiha, Adi Haqi, Kedamay Weyane, and Hawelti, respectively, with no statistically significant difference (*P* > 0.05) between the study sites and parasite occurrence.

Under the current study, management type was found to have a statistically significant difference (*P* < 0.05) with strongyle infection in horses and donkeys revealing infection rates of 60% and 39% for extensive and semi-intensive management systems, respectively. However, the current study has reported a lower infection rate than that reported by Mezgebu et al. [27], 83.33% and 80.43% in grazing and mixed feeding, respectively, in and around Gondar town, which could indicate that the prevalence of extensive management system is higher than semi-intensive management system, which could be related to husbandry practices, agroecology, hygienic condition, deworming services, study design, sample size, and analysis approach.

## 5. Conclusion and Recommendations

Strongyle infections are important health problems of equines in the study areas with an overall prevalence of 53.13%. Factors such as species, age, body condition, and management type were found to be contributing risk factors in the parasite infection rate, whereas sex, species, and origin of study animals were found to have nonsignificant difference with the parasite infection. In order to minimize losses attributed to the equine strongyle infection in the area, equine owners should be informed of the economic importance and methods of control and prevention of helminths of equines through management improvements. Furthermore, research should be encouraged on equine strongyle parasites and regular and strategic deworming programs, and efficacious anthelmintics should be administered regularly. Moreover, advanced epidemiological studies on equine strongyle need to be strengthened.

## Figures and Tables

**Table 1:** Prevalence and risk factor analysis results.

Risk factor	Number of examined	Number of positive	Prevalence	COR (95% CI lower-upper)	*P* value
*Species*					
Donkeys	200	106	53		0.959
Horses	184	98	53.3	0.990 (0.663–1.478)	

*Sex*					
Male	351	189	53.85		0.357
Female	33	15	45.5	1.4 (0.684–2.867)	

*Age*					
Young	48	20	41.7		0.003
Adult	206	126	61.2	0.454 (0.240–0.859)	
Old	130	58	44.62	0.88 (0.45–1.73)	

*Body condition*					
Good	130	57	43.85		*P* ≤ 0.001
Medium	166	75	45.18	0.947 (0.597–1.504)	
Poor	88	72	81.82	0.174 (0.091–0.330)	

*Management*					
Extensive	265	158	59.62		*P* ≤ 0.001
Semi-intensive	119	46	38.66	2.343 (1.50–3.65)	

*Areas (sites)*					
Hadnet	103	52	50.49		0.291
Quiha	89	42	47.19	1.141 (0.647–2.013)	
Adi Haqi	62	37	59.68	0.68 (0.36–1.30)	
Kedamay Weyane	59	37	62.71	0.606 (0.31–1.16)	
Hawelti	71	36	50.70	0.991 (0.541–1.815)	

Total	384	204	53.13		

**Table 2:** Portrayal of logistic regression analysis results.

Variable	Test result		Bivariate logistic regression		Multivariate logistic regression	
	Positive	Negative	COR (95% CI)	*P* value	AOR (95%CI)	*P* value
*Age*						
Young	20	28	1		1.063 (0.510–2.213)	0.871
Adult	126	80	0.454 (.240–0.859)	0.015	0.370 (0.225–0.611)	*P* ≤ 0.001
Old	58	72	0.887 (0.454–1.733)	0.725	1	

*Body condition*						
Poor	57	73	1		6.990 (3.542–13.793)	*P* ≤ 0.001
Medium	75	91	0.947 (0.597–1.504)	0.819	6.131 (3.191–11.781)	*P* ≤ 0.001
Good	72	16	0.174 (0.091–0.330)	*P* ≤ 0.001	1	

*Managements*						
Extensive	158	107	1		1	
Semi-intensive	46	73	2.343 (1.505–3.65)	*P* ≤ 0.001	2.552 (1.571–4.147)	*P* ≤ 0.001

## Data Availability

The analysed data can be released if it is necessary and requested by the concerned body. For time being, for data security/copyright/safety purpose, the data are unavailable. So, the data used to support this study are available from the corresponding author upon request.

## Abbreviations

AOR: Adjusted odds ratio
CI: Confidence interval
COR: Crude odds ratio
CSA: Central statistic authority
TBOANR: Tigray Bureau of Agriculture and Natural Resources
GI: Gastrointestinal.
